# A Qualitative Study Exploring the Role of Pharmacists in Medical Student Training for the Prescribing Safety Assessment

**DOI:** 10.3390/pharmacy6030087

**Published:** 2018-08-21

**Authors:** Fay Al-Kudhairi, Reem Kayyali, Vilius Savickas, Neel Sharma

**Affiliations:** 1 Department of Pharmacy, University Hospital Lewisham, Lewisham High St, London SE13 6LH, UK; fay.al-kudhairi@nhs.net; 2 Department of Pharmacy, Faculty of Science, Engineering and Computing, Kingston University, Kingston-Upon-Thames KT1 2EE, UK; viliussavickas@gmail.com; 3 Division of Gastroenterology and Hepatology, National University Hospital Singapore, 5 Lower Kent Ridge Rd, Singapore 119074, Singapore; drneelsharma@outlook.com

**Keywords:** inter-professional, education, pharmacist, medical, undergraduate, PSA

## Abstract

Five years after the introduction of the Prescribing Safety Assessment (PSA) in the UK, the role pharmacists play to help prepare medical students for this challenge is uncertain. Our study explored pharmacists’ perceptions about their role in undergraduate medical training for the Prescribing Safety Assessment (PSA). One hundred and seventy-nine prospective participants from UK hospitals and education and training boards were emailed an interview schedule aimed at ascertaining their current involvement in undergraduate medical education, particularly the preparation for PSA. Responses received via email were thematically-analysed. A total of 27 hospital pharmacists and 3 pharmacists from local education and training boards participated in the interviews. Pharmacists were positive about their involvement in medical student training, recognising the added value they could provide in prescribing practice. However, respondents expressed concerns regarding resource availability and the need for formal educational practice mentoring. Despite a low response rate (17%), this research highlights the potential value of pharmacists’ input into medical education and the need for a discussion on strategies to expand this role to maximise the benefits from having a pharmacist skill mix when teaching safe prescribing.

## 1. Introduction

An increasing amount of evidence suggests a positive reception of pharmacist-led inter-professional education (IPE) amongst medical undergraduates leading to an enhanced understanding of their roles within the multidisciplinary team and an ability to identify medication-related problems [1,2]. Similarly, pharmacist-led postgraduate training of doctors results in improved prescribing practice and medication safety [3,4]. Despite this, little is known about the extent of pharmacists’ involvement in the education of their junior medical colleagues.

The need to explore the role of pharmacists in undergraduate medical education has intensified in recent years with increasing concerns over the prescribing competence of foundation doctors, in principle raised by the EQUIP study [5]. The development of the Prescribing Safety Assessment (PSA) [6] and the new undergraduate medical curricula [7], which aimed to address some of these concerns, created a further need to involve pharmacists in the preparation of the next generation of doctors.

A scoping questionnaire for former medical students revealed that 9 out of 10 respondents valued pharmacist-led training, which they felt would have supported their prescribing and preparedness for the PSA (data not shown). In turn, this study aimed to explore pharmacists’ perceptions about their current involvement in the education and training of medical undergraduates in preparation for the PSA.

As the great majority of undergraduate clinical placements take place in the hospital environment [8], pharmacists working in secondary and/or tertiary care may be ideally placed to facilitate medical student education and preparedness for practice. Therefore, this qualitative study primarily targeted UK hospital pharmacists who might have the greatest amount of contact time with medical undergraduates.

## 2. Materials and Methods

A convenience and snowballing sampling strategy was used to recruit prospective participants. The contact details of prospective participants were obtained by one of the researchers via the Pharmalife website, which at the time facilitated the recruitment of pre-registration trainee pharmacists and contained the email addresses of the Lead Education and Training Pharmacists in each NHS Trust in England and Wales. The Education and Training Pharmacist in each Trust acted as a gatekeeper to snowball an email invitation to participate in an interview within their respective pharmacy department. The first author of the manuscript then approached individuals who expressed an interest to participate by email, providing them with a participant information sheet and an interview schedule. Participant’s email response to questions in the interview schedule constituted an implied consent to take part.

A total of 176 pharmacists from National Health Service (NHS, UK) Trusts and three education and training pharmacists from local education and training boards (LETBs) across the UK were sent an email invitation to participate in semi-structured email interview. The choice of this interview method was preferred by interviewees over telephone or face-to-face alternatives due to increased flexibility and was expected to maximise the response rate [9].

The interview schedule consisted of 14 open questions designed to ascertain the perceived role of pharmacists in the education of medical undergraduates (Appendix A). Pharmacists were also asked about their knowledge related to the PSA and any impact this assessment might have had on their role in undergraduate medical education in order to support final-year medical students undertaking this assessment. The interview schedule was piloted with two pharmacists working within the academic institute who had education and training roles in NHS Trusts with minor changes. Thematic analysis of qualitative data was conducted using a 6-step method adapted from Braun and Clarke [10]. Analysis was carried out in a constant comparative manner until data saturation was reached. The stopping criterion for data saturation, which relates to the number of interviews conducted in the absence of any new data emerging, was six. Data saturation was reached after the 24th interview. One member of the research team transcribed the data, whilst two members were involved in the analysis including the coding and subsequent comparative analysis to identify any themes. The trial coding of the text involved assessing the accuracy and reliability of the coding procedure. As no disputes were found, the coding was maintained for the rest of the text, and conclusions were derived from the coded data.

## 3. Results

Twenty-seven pharmacists (all from different NHS Trusts) and all three pharmacists from LETBs took part in email interviews. The majority of respondents specialised in education and training of either or both other health care professionals (HCPs) and/or pre-registration trainee pharmacists (*n* = 10/27). Six respondents were either deputy chief, lead, highly specialist, or advanced pharmacists in their respective areas. The remainder of pharmacists specialised in other areas of clinical pharmacy, such as renal or critical care.

All pharmacist participants were asked to identify the advantages and limitations of pharmacists’ involvement in the education and training of other HCPs (Table 1). The results derived from this question demonstrated that pharmacists believed their educational role had a positive impact on other HCPs, students, themselves, and patients. An emphasis was placed on pharmacists being experts in medicines who “can offer a unique perspective to teaching” and that, through regular interactions with other HCPs, pharmacists can “identify common errors to target future training.” This in turn may lead to “improved basic knowledge [of prescribing], which improves patient safety.” One participant felt that improved knowledge of medicines amongst junior doctors may also free up pharmacists’ time traditionally used to answer medicines-related queries and would enable them to conduct “more specialised work and interventions”, which would ultimately enhance patient care.

Some pharmacists, however, did not feel supported enough to carry out training, either because “pharmacists do not routinely receive training on how to provide educational sessions” or due to a “lack of adequate resources and support from the organisation”. Participants thought that such barriers may be overcome by “delivering educational sessions only where their expertise is called upon” and that certain sessions may be more appropriately delivered by experts from a different discipline.

**Figure 1:**
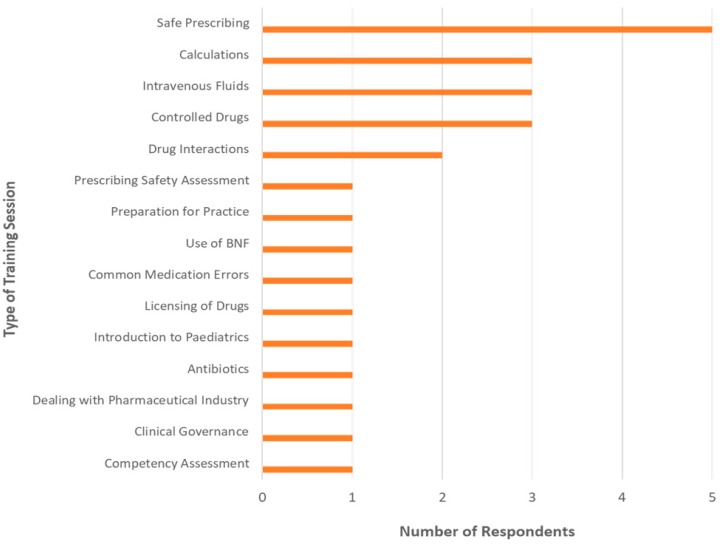
A range of training sessions delivered by pharmacists at the participating NHS Trusts (*n* = 14/27, excluding pharmacists from Local Education and Training Boards).

All 27 hospital pharmacists indicated that either themselves or other pharmacists at their hospital were involved in the active education of other HCPs. However, in only 14 Trusts this involvement extended to medical undergraduate education. A range of pharmacist-led training sessions for medical students were listed by participants with safe prescribing as the most popular focus followed by controlled drugs, intravenous fluids, and calculations (Figure 1). Pharmacist participants anticipated that the training delivered to medical undergraduates “impacted on their practice and hopefully made them better prescribers in the future”.

When asked if they had heard of the PSA, 23 said they had, with 12 either becoming more involved in teaching general therapeutics and prescribing to undergraduate medical students, or if already teaching medical students, tailoring their teaching to become more PSA-orientated as a result of the introduction of the assessment. When provided with an outline of the PSA and the associated competencies, all of the respondents agreed that pharmacists should be involved in educating medical students in preparation for the PSA. As “experts in medicines”, pharmacists perceived themselves as “ideal HCPs” to teach medical undergraduates about the safety of prescribing. One pharmacist further added that “pharmacist-led teaching should not be focused only on the [prescribing safety] assessment, but rather the skills needed for future prescribing practice.”

## 4. Discussion

This study aimed to highlight the role of pharmacists in enhancing the education of medical students in preparation for the PSA in the UK. Pharmacists expressed their beliefs about the benefits of their involvement. This focused predominantly on enhancing patient safety through appropriate prescribing knowledge delivery and the skillset they could transfer to medical students. Such perceptions about the significance of pharmacist’s role were not unexpected considering the previous studies, which demonstrated an improvement in the quality of junior doctor prescribing following pharmacist-led educational interventions, thereby potentially leading to improved patient safety [3,4].

Concerns were raised, however, about the lack of resources available for pharmacists to assist fully with time and funding pressures being cited. Furthermore, pharmacists alluded to the fact that in order to teach effectively, formal understanding of educational practice is also necessary. While several courses have been made available to facilitate the development of pharmacist’s educational skillset [11,12], it is clear that such courses may need to be made more widely available and flexible in order to accommodate the role of practicing pharmacists in undergraduate medical education, including the student preparation for the PSA.

At a time where health professional educators recognise the importance of IPE and the science of teaching, this study emphasised concerns that in reality there are notable factors that still need addressing. Some of these factors, for instance the lack of time and resources, may be partially addressed through better utilisation of nationally available e-learning courses for medical undergraduates and junior doctors (e.g., SCRIPT) [13]. The completion of such courses may be followed by discussion or feedback from the designated pharmacist facilitator—an educational model that is known to be effective in practice [4].

Our study findings are in contrast to existing data, which emphasised the existence of pharmacist-led teaching sessions on the wards, either one on one or in a group settings, as well as their involvement in clinical pharmacology and therapeutics [14,15].

## 5. Conclusions

Due to various barriers, few of the pharmacists in our study were actively involved in medical student education, yet respondents were generally positive about increasing their participation in prescribing-related training. Whilst we recognise the small sample size, we hope that our findings help to ensure that medical students’ prescribing knowledge benefits from their pharmacist colleagues, and that they are supported adequately in their teaching endeavours.

## Figures and Tables

**Table 1:** A summary of themes relating to advantages and limitations of pharmacists’ involvement in the training and education of other HCPs identified through the analysis of qualitative interview data (*n* = 30). Abbreviations: CPD—continuing professional development; IPE—inter-professional education; HCPs—healthcare professionals.

Advantages	Limitations
Pharmacists’ specialist knowledge in medicines	Not all pharmacists are teachers by nature/no formal training
Pharmacists’ perspective on patient, not disease, attention to detail	Teaching may have limited perspective and not be multi-disciplinary focused
Improved patient safety and care	Time taken from usual work commitments
Raises profile of pharmacists	Need dedicated teaching role in order to ensure compliance with sessions/appropriate follow up
Contributes to pharmacists’ CPD	Pharmacists’ lack of medical knowledge/medical experience
Encourages IPE between HCPs	Lack of awareness of pharmacists’ knowledge and skills by other HCPs
Medical students benefit from practical knowledge of prescribing	Lack of funding and resources as support

## Appendix A. Interview Schedule

Project student: Fay Al-Kudhairi

Email: k1001441@kingston.ac.uk

Supervisor: Reem Kayyali

Email: R.Kayyali@kingston.ac.uk

Dear Pharmacist,

I would like to invite you to take part in this interview, which aims to ascertain your opinions and knowledge regarding pharmacists’ role in educating junior doctors and other healthcare professionals, and what, if any, interprofessional (IPE) activities pharmacists in your hospital/trust are involved in.

For the purpose of this interview, IPE is defined as different professionals learning with, from, and about each other to strengthen collaboration and improve the quality of care provided, including professionals working with healthcare students.

Studies have shown the benefits of pharmacist-led teaching of healthcare professionals, especially in terms of reducing prescribing errors. However, there are no studies that identify the range of teaching of other healthcare professionals that takes place by pharmacists.

Please answer the following questions as best you can. This interview should take no more than 15 min to complete.

1. Are any of the pharmacists at this hospital involved in the active education of healthcare professionals (HCP), other than everyday sharing of knowledge on the wards as part of normal practice such as workshops, training days, or seminars? If yes, go to Q2. If no, go to Q4.
2. Please can you provide more information on the activities, i.e., what types of activities are there? Which HCPs are taught? How often are sessions given? Which pharmacists run the sessions? Which topics are taught? Is there feedback on the activities? Is the teaching based on any specific model of education and training?
3. Do pharmacists at this trust run sessions for HCPs at other hospitals? If yes, please specify where.
4. Please state the reasons/possible barriers why no pharmacist-led teaching occurs at the hospital and suggest how these barriers can be overcome.
5. What would you say are some of the major benefits of pharmacist-led teaching to all HCPs?
6. What, in your opinion, are the disadvantages/limitations of education sessions run by pharmacists, if there are any?
7. Can you give an example of when you have run an educational session for other HCPs and explain what impact it had on them and their practice?
8. Do pharmacists in this hospital receive training from other healthcare professionals? Please provide brief details.
9. What do you predict for the future of pharmacists as educators of other pharmacists and other HCPs? Is your prediction based on any specific models of education and training?
10. Have you heard of the Prescribing Safety Assessment (PSA) for final year medical students? (If no, please go to Q12)
11. If you answered yes to question 10, please specify what, if any, changes to or introduction of pharmacist-led teaching to medical undergraduate students have occurred as a result of the introduction of the PSA?
12. In response to studies that show the prescribing error rates among junior doctors, as well as how unprepared medical students feel regarding prescribing after graduation, the British Pharmacological Society and the Medical Schools Council created an assessment, the PSA, to try to overcome these issues and provide a reliable assessment of students’ prescribing skills that is now a requirement for all UK final year medical undergraduates to undertake. The assessment consists of eight sections, each assessing key competencies for junior doctors: adverse drug reactions, calculation skills, communicating information, data interpretation, drug monitoring, planning management, prescribing, and prescription review. The assessment also tests skills in use of the BNF. Each of these competencies can be related to the seven key clinical domains: elderly care, general practice, medicine, obstetrics and gynaecology, paediatrics, psychiatry, and surgery. In light of this information, do you believe pharmacists could have a role in supporting medical students with this assessment? Please explain your answer.
13. Is there any other information you think may be useful for me to know? Please attach any relevant documents or provide links below to relevant websites to support the information you provided, if appropriate.
14. Do you have any questions?

Thank you for taking part in this interview. Your participation is much appreciated. I would be grateful if you could return this completed questionnaire to k1001441@kingston.ac.uk at your earliest convenience.
